# Sound Stabilizes Locomotor-Respiratory Coupling and Reduces Energy Cost

**DOI:** 10.1371/journal.pone.0045206

**Published:** 2012-09-27

**Authors:** Charles P. Hoffmann, Gérald Torregrosa, Benoît G. Bardy

**Affiliations:** 1 Movement to Health (M2H) Laboratory, EuroMov, Montpellier-1 University, Montpellier, France; 2 Institut Universitaire de France, Paris, France; McMaster University, Canada

## Abstract

A natural synchronization between locomotor and respiratory systems is known to exist for various species and various forms of locomotion. This Locomotor-Respiratory Coupling (LRC) is fundamental for the energy transfer between the two subsystems during long duration exercise and originates from mechanical and neurological interactions. Different methodologies have been used to compute LRC, giving rise to various and often diverging results in terms of synchronization, (de-)stabilization via information, and associated energy cost. In this article, the theory of nonlinear-coupled oscillators was adopted to characterize LRC, through the model of the sine circle map, and tested it in the context of cycling. Our specific focus was the sound-induced stabilization of LRC and its associated change in energy consumption. In our experimental study, participants were instructed during a cycling exercise to synchronize either their respiration or their pedaling rate with an external auditory stimulus whose rhythm corresponded to their individual preferential breathing or cycling frequencies. Results showed a significant reduction in energy expenditure with auditory stimulation, accompanied by a stabilization of LRC. The sound-induced effect was asymmetrical, with a better stabilizing influence of the metronome on the locomotor system than on the respiratory system. A modification of the respiratory frequency was indeed observed when participants cycled in synchrony with the tone, leading to a transition toward more stable frequency ratios as predicted by the sine circle map. In addition to the classical mechanical and neurological origins of LRC, here we demonstrated using the sine circle map model that information plays an important modulatory role of the synchronization, and has global energetic consequences.

## Introduction

In order to produce the mechanical energy vital to move, the organism of humans and other animals must furnish to the muscles the chemical energy that they need. Respiration does the job, and brings dioxygen (O_2_) from the environment into the organism. One fundamental issue raised by the requirement of breathing during moving is thus the subtle coupling between the respiratory system and the locomotor system, a phenomenon known as the Locomotor-Respiratory Coupling (LRC). Several studies [1]–[4] have reported a natural synchronization between respiration and locomotion in many species (mammals, fish, birds) and for various forms of locomotion (running, cycling, rowing, wheelchair propulsion). Generally, entrainment between respiration and locomotion has been described by stable frequency mode locking patterns (i.e. ratio of breathing cycles over locomotion cycles) such as the ones reported in humans: 1/1, 1/2, 2/3, or 1/4 [1]. LRC is generally understood as originating from mechanical and neurological interactions. From the point of view of mechanics, respiratory entrainment is the result of the impact loading on the thorax as the limbs strike the ground (the visceral piston), using common respiratory and locomotor muscles (i.e., abdominal muscles), spinal flexion and extension, body acceleration and deceleration in horizontal and vertical planes [1], [5]. The visceral piston is especially at work during bipedal locomotion, such as walking and running. However, the upright posture in humans permits to reduce the mechanical constraints imposed by locomotion on respiration and allows larger ventilatory adaptations than for other mammals, quadrupeds for instance. Lee & Banzett [5] showed that the mechanical contribution of locomotion to tidal volume during walking and running is around 2% in humans. In cycling, the mechanical constraints between the locomotor and respiratory systems (visceral piston or spinal flexion/extension) are even more reduced compared to running, but nevertheless exhibit LRC. Thus, the observed synchronization between respiratory and locomotor systems, in spite of weak mechanical interactions [6], suggests that other mechanisms are operating. Neurological interactions have been offered as a complementary origin of LRC [7], [8], but their role has not yet been confirmed in humans. However, several animal studies have shown a common command for respiration and locomotion at both cortical [9] and medullar levels [10]. Direct interactions between respiratory and locomotor central pattern generators were clearly observed in decerebrated animals [11]. It thus appears that LRC in vertebrates results from the combination of multiple peripheral and central mechanisms, themselves modulated by exogenous variables (see below), which contribution is largely unknown. The LRC literature reveals serious discrepancies related to the nature of the coupling between the locomotor and the respiratory systems, the factors that modulate it (movement frequency, workload, skill level, *etc.*), and the concomitant energy consumption (see below for details). These contradictory results seem in part due to various definitions of the synchronization phenomenon and different methodologies used to assess LRC. In this article, we viewed LRC as the result of non-linear coupled-oscillators, captured by the sine circle map model, influenced by external factors such as information, with consequences on global energy consumption. We offered a rigorous method to compute LRC during various motor situations, using four related variables extracted from the sine circle map model. We tested the stabilizing effect on LRC of an external rhythmic stimulation, and evaluated the accompanying energy consumption.

### LRC, Expertise, and Energy Consumption

Bramble & Carrier [1] reported an effect of expertise on LRC during running. They found more entrainment between locomotion and respiration in experts than in novices. This result was confirmed in other sports such as cycling [12] or rowing [13]. In contradiction, McDermott, Van Emmerick & Hamill [14] observed no changes in LRC related to the degree of expertise. They concluded that training had no effect on LRC occurrence. However, they suggested a qualitative solution used by experts to adapt LRC, in the form of a transition from the modal frequency ratio to other ratios. Bernasconi *et al.* [15] confirmed this suggestion. They assessed the degree of coordination – defined in their case as the maximal percentage of combined inspiration and expiration cycles starting in the same phase of step previously divided into ten bins – for three groups of participants (triathletes, sprinters and controls) and at three exercise intensities (50%, 80%, and 110% of the anaerobic threshold). They demonstrated that coordination increased in all groups with increasing intensities (from 50% to 80%). However, only triathletes were able to maintain their degree of coordination when the intensity reached 110%. Hence, the type of training in aerobic intensities was related to the ability to coordinate locomotion and respiration above the anaerobic threshold. This result points to the fundamental but unclear role of LRC in energy transfer. Bramble & Carrier [1] suggested that a greater LRC could lead to a decrease in O_2_ consumption (VO_2_). This assumption was investigated by several authors, occasionally confirmed [2], [16], [17], but with exceptions [18]–[21]. This discrepancy in the literature may be due to (i) the small decrease in VO_2_ found with stable LRC values, (ii) the difference in workload or populations, or as evidenced below, by (iii) the contribution of other uncontrolled variables.

### Informational Influence on LRC

In bimanual coordination, the *anchoring effect* describes the effect of an external stimulation on the coupling between the two hands. Fink *et al.* [22] and others [23] showed that a periodic auditory stimulation stabilizes both in-phase and anti-phase coordination (local stabilization) and postpones the transition from anti-phase to in-phase (global stabilization). This effect persists when finger movements are synchronized with complex rhythmic sequences [24]. Without evoking the *anchoring effect*, Haas *et al.* [25] reported a greater stabilization of the respiratory rhythm under a rhythmic external auditory stimulation even if participants had no instruction concerning their respiratory frequency. In addition, they reported a greater stabilization of the respiratory rhythm when rhythmic tapping movements were added to the task, suggesting that when a movement is coordinated with an auditory stimulation then the respiratory rhythm is more regular. Other LRC studies have found similar results, with more entrainment between locomotion and respiration when a metronome was used to pace the locomotion frequency [2], [17], [26]. Thus, an external (auditory) stimulus seems to be an efficient way to stabilize LRC.

### Circle Map and Farey Tree

We pointed out several discrepancies in the LRC literature originating from the use of different methodologies. Our method uses the non-linear coupled-oscillators model showing that when two oscillators with different eigenfrequencies are coupled, their interaction results in attraction to a certain frequency ratio, which depends on the ratio between the eigenfrequencies (i.e., the *bare winding number* Ω) and the strength of the coupling of the oscillators (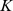). A coupled-oscillators system is often considered as the general case of a single-oscillator system periodically forced by an external force [27]–[29]. One particular output of this model for LRC is the observation of how often the phase of one oscillator (the forced oscillator) at discrete time intervals corresponds to each cycle of the other oscillator (the forcing oscillator). Circle maps describe the behavior of the phase 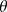 of the forced oscillator measured at each periodic time interval 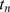 of the forcing oscillator via the following iterative equation:
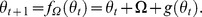



This equation maps the state of the forced oscillator onto itself from three factors: the phase at the previous cycle (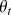), the ratio between the eigenfrequencies of the uncoupled forced and forcing oscillators (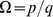), and a nonlinear strength function (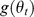), a periodic force influencing the iteration of 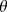. The coupling function 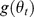 is usually defined by a sine function 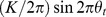, where 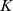 represents coupling strength.

**Figure 1:**
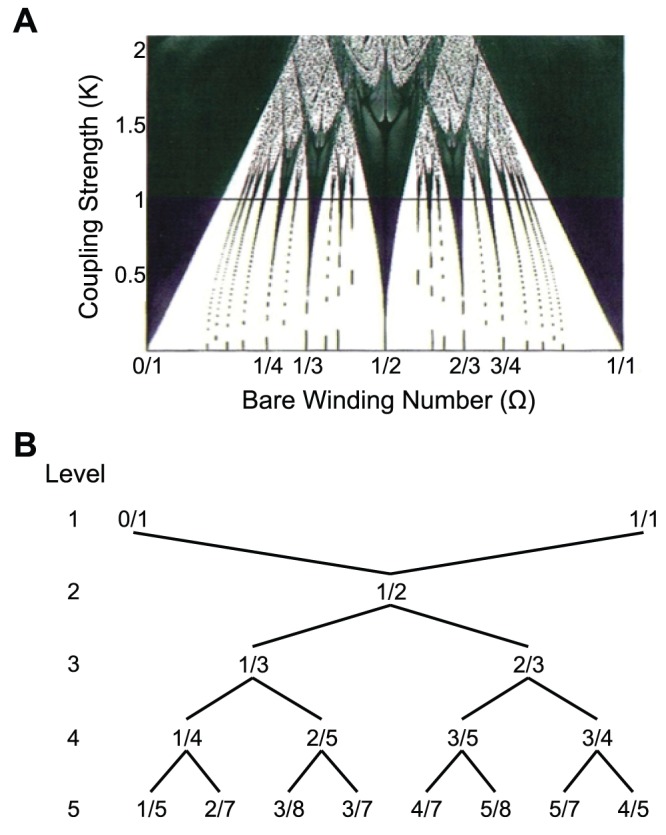
Representation of Arnold Tongue and the first five level of the Farey tree. A. Regime diagram: For a particular coupling strength (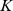), specific mode locks occur within ranges of 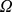
(the ratio P/Q when the oscillators are uncoupled: the ‘bare’ winding number). The larger a particular range, the more stable is the associated mode-locked ratio. Arnold’ tongues for ratios up to Farey level 8 are displayed. Darker regions correspond to more stable behavior (Reprinted from “Springer and Biological Cybernetics, Volume 73 (1995) 301–309, Peper *et. al.*, Original Copyright (c) 1995, Springer Publishing Company, LLC”, with kind permission from Springer Science and Business Media). B. Five first levels of the Farey tree: this hierarchical mathematical structure corresponds to the organization of frequency ratios as predicted by the sine circle map. Lower-level frequency ratios are more stable than higher-level ratios.

In order to evaluate if the two oscillators adjust their eigenfrequencies to each other, one can examine how an external force influences the forced oscillator after a great number of rotations, and guides it toward the synchronization region. The iteration of the map is usually described by the *winding number* 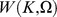.
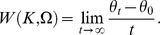
where 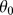 corresponds to the initial phase of the forced oscillator. 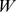 represents the mean number of rotations of the phase per period of the external force. The *winding number* predicts the frequency ratio observed after a great number of rotations. All 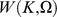 depending on the variation of 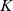 and 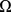 correspond to the region of synchronization between the two oscillators and can be represented by the Arnold tongues diagram (Figure 1A). On Figure 1A, darker regions correspond to rational 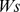 (indicating resonance or frequency locking). The tongues width allows classifying resonance regimes by their stability. For instance, higher-order Arnold tongues (narrowest tongues, consisting of largest numerators and denominators) permit a mode locking only when 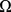 is weak or when 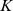 is strong. For biological systems, it implies that natural noise or ubiquitous perturbations can induce transitions from a narrow to a wider Arnold tongue, as demonstrated for coordinated rhythmic hand movements [28], [30], [31]. The sine circle map model predicts a hierarchy of frequency locking modes depending on the initial conditions of 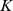 and 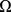. Two major regions in the regime diagram can be observed. If 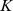 <1, the periodic tongues do not overlap, opening the possibility for 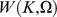 to be irrational. In this case, the behavior of the forced oscillator is quasi-periodic. Rational 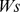 are still present but they are so narrow that quasi-periodic behaviors may occur. If 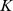 ≥1, periodic tongues overlap and only rational 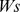 are available. If the forced oscillator stays in a region of synchronization, the behavior is periodic. Consequently, the forced oscillator can transit from one to another tongue and a chaotic behavior may be observed [32].

Even if the sine circle map is an elegant model to understand the coordination between two coupled oscillators, its use for the experimental characterization of LRC remains very difficult. The Farey tree is a simplified way to represent the coordination expressed by the Arnold tongues regarding the stability of 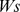 [28]. The Farey tree (Figure 1B) is a mathematical structure organizing the rational integer ratios by levels, where each level reflects the relative stability of frequency ratios as predicted by the sine circle map. For instance, 1/2 is predicted to be more stable than higher order frequency ratios such as 1/3 or 2/3. Figure 1B represents the first five levels of the Farey tree. If we consider two parent frequency ratios (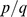 & 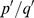), the median ratio between the two parent ratios is defined by:
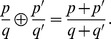
where 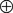 represents the Farey sum. For instance, the lowest level (Level 1) contains the parent ratios 0/1 and 1/1 of which the Farey summation produces the frequency ratio 1/2 at Level 2. The systematic application of the Farey summation can be used to generate the entire tree of ratios. This model has two major properties: it allows classifying hierarchically the observed frequency ratios by their stability predicted by the circle map model, and it provides precise boundaries for the determination of observed frequency ratios. This second property, although never analyzed in the LRC literature, is of crucial importance because it severely impacts the observed results. It particularly retained our attention in the present study.

### The Present Study

Several authors have used the sine circle map model to better capture complex human bimanual coordination. For instance, Peper *et al.* [28] studied hand coordination in drummers and evidenced that the circle map provides a useful general mathematical model to capture multi-frequency mode-locking behaviors. Indeed, they imposed a particular frequency ratio to participants and observed a loss of stability when frequency increased, leading to transition toward more stable regions of synchronization [31]. Studies by Temprado *et al.* [6], Villard *et al.* [33] and Gonzales *et al.* [34] suggested that the nonlinear coupled-oscillators model could also be applied to study LRC. Hessler *et al.* [35] observed a stability difference in arm-respiration multi-frequency ratios, with ratios of low-order levels in the Farey tree being more stable than ratio of higher-order levels. However this model was essentially used in tasks where the energetic component of the movement is very limited. The present study tests the validity of the circle map model to assess LRC, its stability, and its flexibility during enduring physical activity. Our first goal was to confirm the existence of LRC using this model, and to evaluate the stabilizing effect of an auditory periodic stimulation. Our second goal was to evaluate whether stronger LRC, defined using the sine circle map model, was accompanied by a decrease in oxygen consumption (VO_2_).

## Materials and Methods

### Participants

Sixteen male athletes (age 24.63±2.47 years, body mass 72.63±12.15 kg, height 177.94±5.28 cm) not specialized in endurance sports participated in the experiment. None reported any health problem and all were enrolled in a sport association. They all signed an informed consent before participating in the experiment, and received a financial compensation of 30€ for their participation. The experiment was approved by the regional ethic review board (*Comité Consultatif de Protection des Personnes de Nîmes Sud-Méditerranée 3*, CPP n°20090705), conforming to the declaration of Helsinki.

### Experimental Setup

Experimental trials were performed on cycle ergometer (Ergoline 800S, Hoechberg, Germany) at the EuroMov centre in Montpellier. Gas exchanges and ventilation were recorded and analyzed continuously breath by breath by an automated system (ZAN 600 Ergo test, ZAN Messgerate GmbH, Oberthukba, Germany). The gas analyzers were calibrated before each test according to manufacture specifications (the ZAN instruction manual) using room air and gas concentrations (16% O_2_ and 5% CO_2_). The air volume was calibrated with a 1-L syringe. Breathing kinematics was measured by a thermocouple sensor (SS6L Temperature Transducer BSL, Biopac Systems Inc., Santa Barbara, USA) placed in front of the mouth into the mask worn by the participant. An electromagnetic sensor located on the left side of the ergometer calculated the pedaling rhythm. It detected the passage of a magnet fixed on the left crank. A pressure sensor was placed in the right shoe of the participant to determine the moment at which the maximum force was exerted on the right pedal. Temperature, electromagnetic and pressure signals were collected simultaneously at 1000 Hz through a data acquisition board (NI USB-6009, National Instruments, Austin, Texas, USA).

### Procedure

The experiment included five sessions, spread evenly over a period of 14 days. In session 1, participants completed a maximal incremental exercise under medical supervision (VO_2_ max), in order to determine their individual power output at their anaerobic threshold (PAT) [36], [37]. In session 2 (FREE Session), after a 3-minutes warm-up, participants performed a 10-minute exercise at PAT. During that session, their individual preferred respiratory (R_fpref_) and locomotor (L_fpref_) frequencies were determined. In sessions 3 and 4, participants performed the same constant-load exercise at PAT in two different conditions: 5 minutes with acoustic stimulation (Sound-On) and 5 minutes without stimulation (Sound-Off). In one of these last two sessions, the periodic externally paced acoustic stimulation was presented at R_fpref_ to the participants who were instructed to exhale (RESP) in synchrony with the stimulation. In the other session, the acoustic stimulation was presented at L_fpref_ and participants were requested to cycle (LOC) in synchrony with the stimulation. Both the 5-minutes periods (Sound-Off or Sound-On) and the synchronization instructions (RESP or LOC) were counterbalanced over all participants.

### Data Analysis and Variables

All analyses presented below were realized using softwares MATLAB 7.10.0 (R2010a) (Copyright © The MathWorks, Inc., 1984–2010) and SCILAB 5.2.2 (Consortium Scilab (DIGITEO), Copyright © 1989–2010 (INRIA), www.scilab.org). First, the respiratory signal was low-passed filtered using a 2^nd^-order Butterworth with a 2 Hz cutoff frequency. After having determined the instant in time of each expiration and pedaling strike, we calculated the frequency ratio for each respiratory cycle. We obtained a time-series of real quotients for each participant in each experimental condition. We used the Farey tree to label the observed frequency ratios through their property to bind any Farey ratio at level n by the adjacent Farey ratios at level n+1 [28]. For instance, for a 2-level Farey tree, five frequency ratios are available: 0/1, 1/3, 1/2, 2/3, 1/1. These ratios are respectively bounded by [0/1, 1/4], [1/4, 1/3], [1/3, 2/5], [2/5, 1/2], [1/2, 2/3] and [2/3, 1/1] intervals. In this example, if the real quotient falls into the [1/4, 1/3] interval (e.g., 0.299), it will be labeled as a ratio of small integers of 1/3. This method implies that lower-order ratios have larger Farey intervals than higher-order ratios. Thus, the chances of labeling a frequency locking as a low-order ratio (e.g. 1/2) are larger than labeling it as a high-order ratio (e.g. 1/4). However, the width of these intervals is representative of the stability difference across frequency ratios defined by the width of the resonance regions (i.e. Arnold tongues) in the sine circle map model [30]. Applying the method for all real quotients, we thus obtained a distribution of Farey ratios. Finally, we used a three-step method to select the most stable level in the Farey tree, corresponding to the closest level to level 1 sufficient to capture the observed frequency quotients. First, we mapped the observed quotient time-series onto 10 Farey trees from 1 to 10 levels, and hence we obtained 10 different distributions of Farey ratios. Second, we determined for each distribution the most frequent Farey ratio (i.e. the distribution mode) and calculated the dispersion of the relative phase between respiratory and pedaling cycle for that mode. Third, the level of the Farey tree leading to the minimal dispersion of relative phase was considered as the most representative to capture the coupling, and therefore the frequency locking between the two systems.

**Figure 2:**
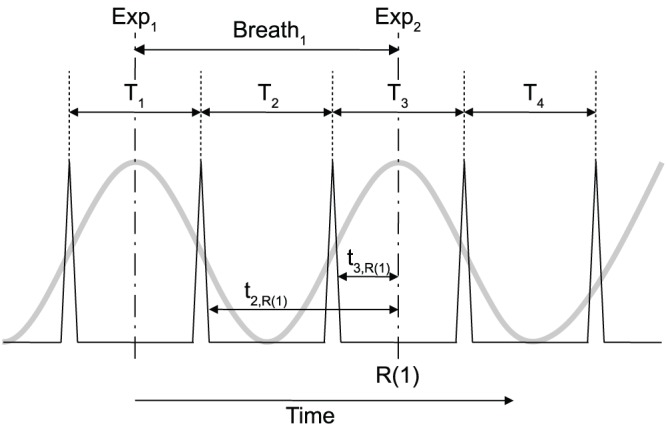
Assessment of the relative phase between breathing and cycling for a 1/2 ratio. The grey curve represents the respiration signal (Exp_i_ corresponds to the beginning of the expiration i) and the black peaks represent the pedalling strikes. 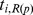 represents the distance from the pedaling strike 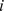 to the end of the p respiratory cycle. 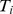 represents the period of the pedalling strike 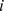.

Formally, the calculation of the relative phase was based on the equation used by previous authors [14] for 1/q ratios but was generalized here to take into account p/q ratios:
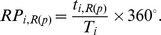
where 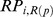 represents the relative phase at the pedaling strike 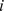 for a p/q mode locking, 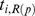 represents the distance from the pedaling strike 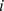 to the end of p respiratory cycle and 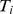 represents the period of the pedaling strike 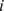 (see Figure 2 for an example).

The equation proposed by McDermott *et al.* [14] only considers the denominator q of the frequency ratio to assess the relative phase between each locomotor cycle and the beginning of the respiratory cycle. However, the sine circle map makes clear that other frequency ratios can be produced, and hence the numerator p of the frequency ratio has to be introduced. p represents the number of respiratory cycles necessary to find the same structure of relative phase time series. For instance, a 1/2 frequency mode locking behavior can correspond to two relative phase values in two different intervals, [0–360°] and [360–720°], repeated for each respiratory cycle resulting in two main bands of relative phase. From the modal frequency ratio previously determined, we can easily plot the relative phase i against the relative phase i+q (the return map) with a lag q correctly chosen. From these maps and following McDermott *et al.* [14], we used a Phase Coupling index (PC) to assess the strength of the coupling between the two systems for the 10 tested Farey trees. The PC index quantifies the dispersion of the relative phase (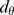) within a tolerance range of ±40 degrees around the identity line on the return map by calculating the Euclidian distance (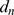) of each point from this line and then summing the weighted distances (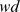).
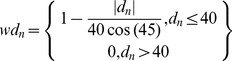


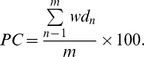
where 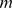 is the number of points of the return map. This measure essentially weights points with distances less than 40° and expresses their sum as a percentage of the highest possible sum. The perfect coupling (represented by PC = 100%) corresponds to all points of the return map lying on the identity line, showing a perfect phase-locking between the two systems. The value of 40° was selected to remain consistent with the value (arbitrarily) chosen by McDermott *et al.* [14].

We also computed the average number of consecutive cycles (in percentage of total cycles) spent on the modal frequency ratio (*LockTime*), as this variable is informative about the local stability of the frequency locking. Finally, a global distribution of the Farey frequency ratios was built for each tested Farey tree representing the mean percentage of occurrence of each Farey ratio including all participants. These distributions were used to assess the modification of the Farey frequency ratios landscape between conditions and evaluate the relative stability of frequency ratios of different Farey tree levels. Finally, an index of stability FRstab (cycles) – corresponding to the ratio between the occurrence of a given Farey ratio and the number of observed transitions from this frequency ratio to another – was calculated to evaluate the resistance of each Farey ratio to transition for each participant in each condition.

**Figure 3:**
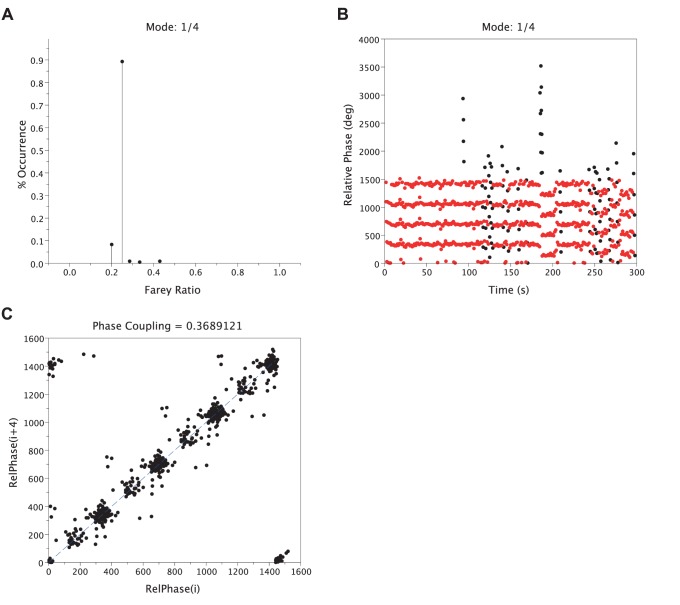
Locomotor-Respiratory coupling (LRC) from one typical participant in one trial. A: modal frequency ratio distribution extracted from the Farey tree (mode = 1/4, level 4). B: 5-minutes evolution of relative phase values where red dots represent the relative phase values for the modal frequency ratio and black dots represent the relative phase values for the remaining frequency ratios. C. Return Map assessing the strength of the phase-coupling by measuring the dispersion of the relative phase values around the identity line.

In sum, to assess the stability of LRC across participants and conditions, five variables were computed, i.e., (i) the percentage of occurrence of the most frequent Farey ratio %*FRmode* (%), i.e., the mode of the distribution (see example in Figure 3A), (ii) the average number of consecutive cycles *LockTime* (%) spent on the modal frequency ratio (see example in Figure 3B), (iii) the phase coupling index *PC* (%) computed as the dispersion of the relative phase between respiratory and pedaling cycles for this mode (see example in Figure 3C) (iv) the global distribution of the Farey frequency ratios (%) and (v) the index of stability *FRstab* (cycles).

### Statistical Analysis

The data were analyzed statistically using the software Statistica 7.1 (Statsoft, France). A two-way repeated-measures ANOVA was performed on LRC variables and concomitant energy expenditure, with sound (Sound-Off vs. Sound-On) and synchronization (LOC vs. RESP) as factors. Post-hoc *Scheffe’s* tests were conducted to determine which conditions were significantly different. The level of significance was set at alpha lower than 0.05 for all tests. A Rayleigh’s test of uniformity was applied to the relative phases distribution between the metronome beat and each pedal stroke (for LOC) or expiration (for RESP) to determine if these distributions were randomly organized or clustered around a mean value.

## Results

The variability of the metronome was around 1 ms. We used a t-test to first verified that the mean frequency of the metronome was not different from the preferred respiratory (RESP) or locomotor (LOC) frequencies assessed during the FREE session for all participants, and that was indeed the case (RESP: t(15) = 0.23, p>.05; LOC: t(15) = 0.19, p>.05). We then tested whether participants followed the instruction to synchronize their breathing or cycling with the metronome. We found unimodal relative phase distributions between each expiration (RESP) or pedal stroke (LOC) and the metronome beat (respectively, 68.14±12.20 and 39.78±61.91 degrees) (all Raleigh tests p<.05 except for one participant p>.05). We thus concluded that all participants but one correctly performed the task.

### Qualitative Analysis

**Figure 4:**
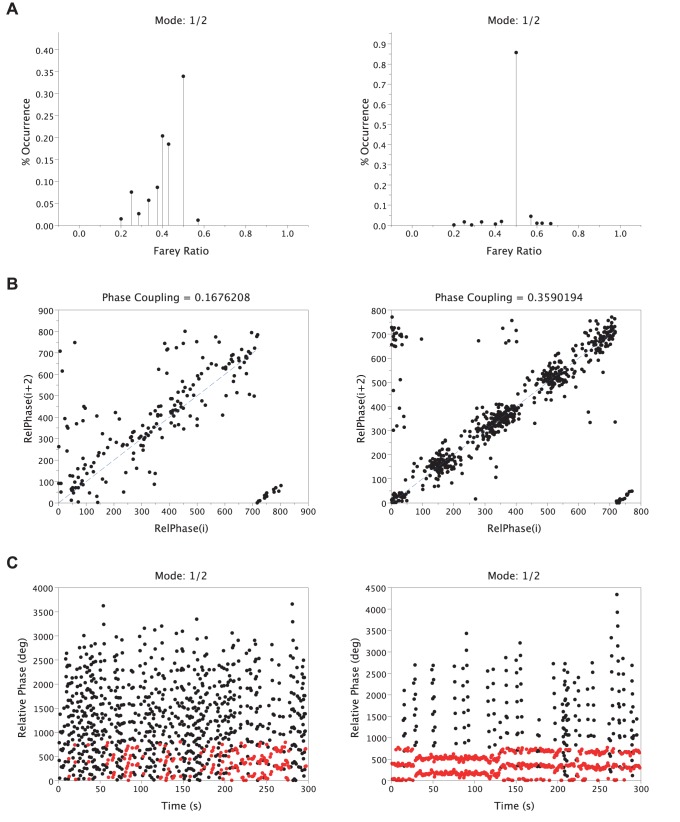
LRC variables for two representative participants in the FREE session. The left three panels correspond to S05 and the right three panels correspond to S15. The first two panels (A. Frequency Ratios Distribution) represent the distribution of the frequency ratios. The next two panels (B. Return Maps) represent the dispersion of the relative phase in the return map. The last two panels (C. Relative Phases) represent the relative phase values during five consecutive minutes where red dots represent the relative phase values for the modal frequency ratio and black dots represent the relative phase values for the other expressed frequency ratios.

**Figure 5:**
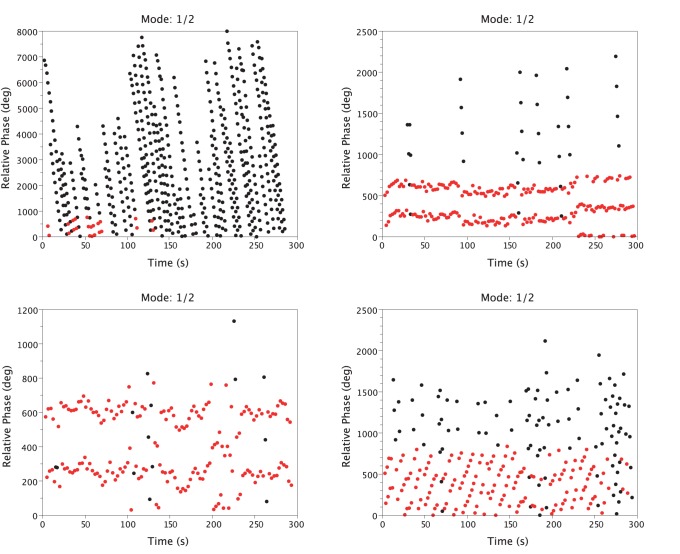
LRC variables for two representative participants in the RESP condition. The left two panels represent three minutes of relative phases for subject S05 (Sound-off at the top and Sound-On at the bottom). The right two panels represent three minutes of relative phases for subject S15. Red dotes represent the relative phase values for the modal frequency ratio and black dotes represent the relative phase values for the other expressed frequency ratios.

We observed a large inter-individual variability on the different variables capturing LRC stability. Figure 4 highlights this variability in the FREE session from two representative participants. Participant S05 spontaneously exhibited a weak LRC, with three main frequency ratios (1/2, 2/5, 5/12, Figure 4A, left), a strong dispersion of the relative phase in the return map (Figure 4B, left) and a weak phase-mode locking with no synchronization band (Figure 4C, left). Inversely, participant S15 spontaneously exhibited a strong LRC with a clear modal frequency ratio (1/2, Figure 4A, right), converging relative phases toward the return map identity line (Figure 4B, right) and a strong phase-mode locking illustrated by the two synchronization bands (Figure 4C, right). Interestingly, the effect of sound on LRC happened to depend on that initial LRC stability. As illustrated in Figure 5 with the same two participants, the sound stabilized (participant S05, Figure 5, left) an initially unstable LRC while it destabilized (participant S15, Figure 5, right) an initially stable LRC.

Performing individual analyses, we found two additional participants resembling S15 above, exhibiting stability reduction in LRC variables with sound, and twelve participants for which sound stabilized LRC variables in both synchronization conditions. Generally, we observed a negative linear correlation between %FRmode in the Sound-Off condition and the difference in %FRmode between both sound conditions (Sound-On - Sound-Off) (RESP R(13) = −0.71, p<.01; LOC R(13) = −0.79, p<.001). We observed similar results for PC (RESP R(13) = −0.77, p<.001; LOC R(13) = −0.75, p = .001) and for LockTime (RESP R(13) = −0.77, p<.001; LOC R(13) = −0.55, p<.05). Together, these results indicate that the stabilizing effect of sound decreased when intrinsic (Sound-Off) LRC stability was high. The three sound-destabilized participants (S02, S15 and S16) were thus distinguished from the other participants on the basis of their behavior during FREE and Sound-Off conditions. Moreover, each of these participants exhibited values of LRC variables (%FRmode, PC and LockTime) higher than the *mean +1 standard deviation* obtained across all participants of our sample.

Following this first analysis, we decided to remove participants S02, S15 and S16 from subsequent analyses. The main results reported in Table 1, which shows mean values and standard deviation for all variables in all experimental conditions, were thus obtained with 12 participants. We performed the same statistical analyses on all 15 participants, revealing similar effects of the auditory stimulation on all variables, for instance VO_2_ (F(1, 14)  = 6.23, p<.05); %FRmode (F(1,14) = 5.07, p<.05;) and PC (F(1,14) = 22.05, p<.001).

**Table 1:** Means ± standard deviation of ventilatory and LRC variables (12 participants).

		Experimental Conditions			
		RESP		LOC	
		Sound-Off	Sound-On	Sound-Off	Sound-On
Ventilatory variables	VO_2_ (L·min^−1^)	2.38±0.37	2.31±0.40	2.35±0.37	2.33±0.36
	VCO_2_ (L·min^−1^)	2.37±0.34	2.30±0.36	2.34±0.36	2.33±0.37
	FR (breath·min^−1^)	29.37±4.85	30.25±4.98	28.91±5.57	31.50±4.19
	StdFR (breath·min^−1^)	2.25±0.82	1.38±0.65	2.32±0.66	2.27±0.87
	VT (L·min^−1^)	2.16±0.30	2.13±0.34	2.19±0.36	2.02±0.32
	VE (L·min^−1^)	62.09±8.50	62.61±9.10	61.50±8.89	62.27±8.24
	VE/VO_2_	24.61±2.68	25.61±3.44	24.74±3.24	24.99±2.25
	VE/VCO_2_	24.65±2.75	25.60±3.17	24.70±3.03	24.96±2.20
LRC variables	%FRmode (%)	56.44±16.03	72.04±16.96	53.39±23.39	67.51±20.10
	PC (%)	12.11±4.25	9.51±6.18	10.21±5.59	18.80±7.93
	LockTime (%)	3.89±1.38	6.70±5.34	4.43±3.03	7.05±5.93
	Level	2±1	1±1	2±1	1±1
	Sound Effect	VO_2_, VCO_2_, StdFR, %FRmode			
	Synchronization Effect	StdFR			
	Sound x Synchronization Interaction Effect	FR, VT, PC			

Values for the Level variable correspond to the median ± inter-quartile interval. All variables significantly affected by the Sound and Synchronization conditions are summarized in the bottom part of Table 1 (p<.05).

#### Ventilatory variables

We noticed a significant reduction of VO_2_ (F(1,11) = 6.10, p<.05) and VCO_2_ (F(1,11) = 7.17, p<.05) with Sound-On, suggesting that the auditory stimulation induced a diminution of energy consumption. A significant sound by synchronization interaction was found on the respiratory frequency (FR) (F(1,11) = 4.14, p<.05), and post-hoc *Scheffe’s* tests revealed that FR increased when participants were instructed to synchronize their pedaling strike with the sound (p<.05). We observed a lower variability of the respiratory frequency (StdFR) in the RESP condition than in the LOC condition (F(1,11) = 8.01, p<.05) and with sound than without (F(1,11) = 10.33, p<.01) but with no interaction between the two factors. Finally, a significant interaction between sound and synchronization was found on the tidal volume (VT) (F(1,11) = 4.85, p<.05). Post-hoc *Scheffe’s* tests revealed (p<.01) that participants reduced their VT when synchronizing their cycling movements with the sound, which compensated for their increase in FR to maintain their minute ventilation (VE) constant. No other significant effect of sound or synchronization was observed, on any other ventilatory variables.

#### LRC variables

We observed a sound-induced increase in %FRmode (F(1,11) = 8.20, p<.05), suggesting that the modal frequency ratio between breathing and cycling was more frequent when paced by the sound, for both synchronization instructions. We also observed a significant interaction between sound and synchronization for PC (F(1,11) = 15.87, p<.01). Post-hoc *Scheffe’s* tests revealed that cycling on the tone increased PC contrary to the other conditions (p = .001). Finally, we observed no significant effect of the two tested factors on LockTime, although a tendency toward an increase when the sound was present (F(1,11) = 4.28, p = .06), in line with the other LRC variables.

**Figure 6:**
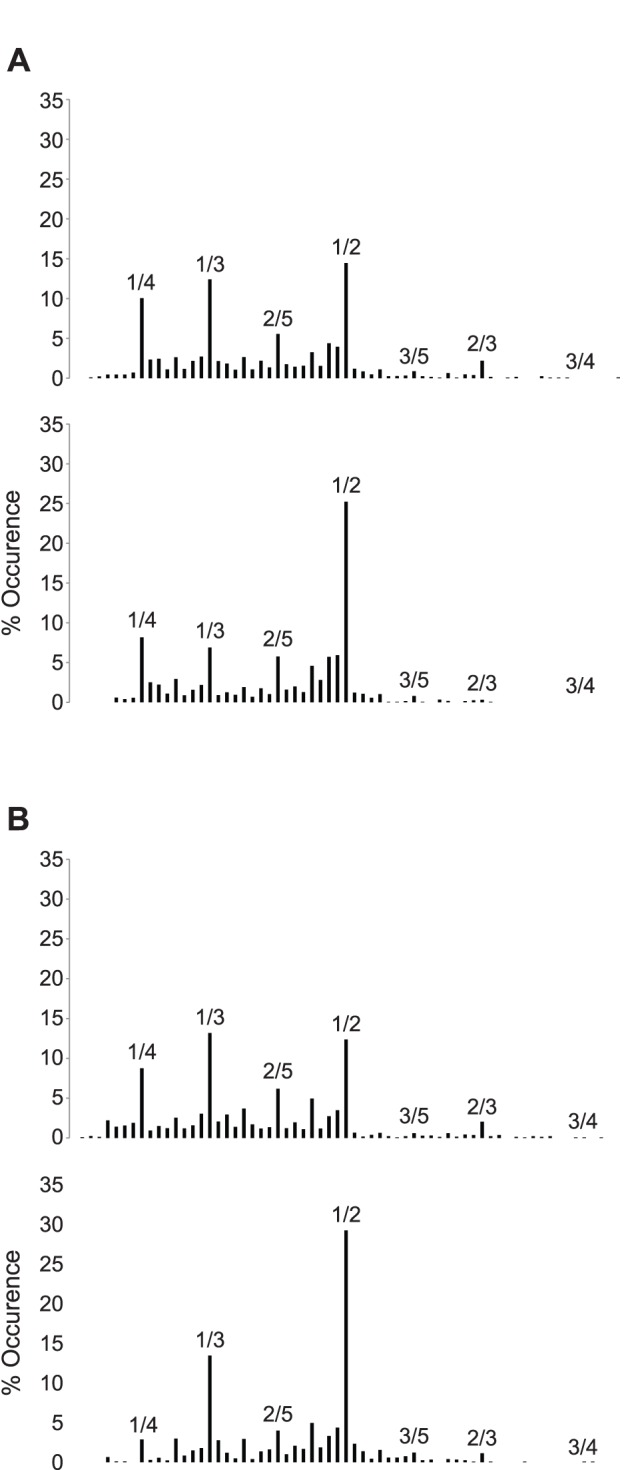
Distributions of the LRC frequency ratios in a six-level Farey tree for the two synchronization conditions. The first two panels represent the RESP condition and the two last correspond to the LOC condition. For each condition the upper panel corresponds to the No-Sound condition and the lower panel corresponds to the sound condition.

Distributions of the LRC frequency ratios: without sound we observed a trimodal distribution in the two synchronization conditions where 1/4, 1/3 and 1/2 occurred more frequently than any other ratios (Figure 6). Then, we analyzed the change of stability of these three frequency ratios when participants were instructed to breathe or to cycle in synchrony with the tone. We observed no main effect of the sound on the occurrence of 1/4 (p>.05) or 1/3 (p>.05) ratios in the two synchronization conditions. We observed a tendency for an increase in 1/2 occurrence (p = .057) when participants breathed in synchrony with the metronome (Figure 6A, bottom), which reached significance (p<.01) when they pedaled in synchrony with the sound (Figure 6B, bottom). Furthermore, the occurrence of the 1/2 ratio became significantly higher than the occurrence of the two other ratios (RESP: 1/4, p<.01 and 1/3: p<.01; LOC: 1/4, p<.001 and 1/3, p<.01) when the sound was present. Taken together, these results suggest that the sound induced a shift in the frequency ratio landscape toward a more stable frequency ratio (i.e., 1/2), in concordance with the other LRC variables. In addition, we observed a gain of stability (FRstab) for the 1/2 ratio with the sound (RESP: p<.05; LOC: p<.05), but a decrease in stability for the 1/4 and 1/3 ratios with the instruction to breeze and cycle in synchrony with the metronome (RESP: 1/4, p<.05 and 1/3, p<.05; LOC: 1/4, p<.05 and 1/3, p<.05).

## Discussion

### The Generalized LRC Model

In this study, we proposed to investigate LRC using the sine circle map, considering the two systems as non-linear coupled oscillators. This method gave us new insights into the LRC dynamics. The first finding is the observation of complex frequency ratios p/q different from the usually 1/q reported ratios. Because the choice of boundaries affects the occurrence of each frequency ratio, it also affects their distribution and limits the comparison between studies. Our main computational novelty is the systematic test of frequency ratios ranked in a hierarchical order in the Farey tree. An important issue not discussed in previous studies concerning LRC is the size of the Farey tree used to assess the observed coordination. Here we proposed a two-steps method: (i) the determination of the modal Farey ratio in a Farey tree composed of 10 levels (from 1 to 10) and (ii) the choice of the level for which the modal Farey ratio exhibits the highest stability, assessed by the phase coupling PC. One other critical point is the labeling of observed frequency ratios. We used here a Farey tree of n+1 level providing precise boundaries to a Farey tree of n level within which each frequency ratio can be rigorously tested (see Data Analysis and Variables) [28], [30]. This procedure ensured to incorporate all rational space in Farey intervals [28]. The difference in interval width affords more probability for low-order ratios to appear and affects the observed distribution of frequency ratios. However, the widths of the Farey intervals are consistent with the relative stability of frequency ratios represented by the widths of Arnold tongues, and represent well the sine circle map dynamics. Our method is inspired from previous work in the area [14] but extends its scope to the case of complex frequency ratios and allows the assessment of frequency-locking modes and relative phase dispersion in LRC. An interesting property of our method is that it allows the simultaneous evaluation of a global index of synchronization (the frequency ratio) together with an index of variability (the phase coupling). The two main results obtained using this method were an informational stabilization of LRC using a harmonic auditory stimulus, and a concomitant decrease in energy expenditure (VO_2_). We discuss these results in turn.

### Sound-induced (De)Stabilization of LRC

In average, the modal value of the frequency ratio %FRmode was 55% without sound and 70% with sound. This result is in line with previous work [15] exhibiting an increase from 56% to 67% of LRC occurrence when participants were instructed to synchronize their breath to an auditory signal related to the footstep, and to the general anchoring effect showing a global stabilization of two coupled systems (i.e. hands) when auditory paced [22]. Other studies [17], [26] reported a similar effect on LRC frequency-mode locking but our study extends the analysis to other variables as well such as phase-mode locking. We observed a positive effect of sound on both frequency-locking and phase-locking variables (%FRmode and PC) when participants were instructed to cycle in synchrony with the metronome. These results suggest a better stabilizing effect of the auditory stimulation on the locomotor system than on the respiratory system. This asymmetry in the sound effect was further validated by the observed change in the respiratory frequency when participants were instructed to cycle in synchrony with the metronome, contrasting with the absence of change in the locomotor frequency when participants were breathing in synchrony with the metronome. Several authors have suggested a unidirectional coupling from the locomotor system to the respiratory system [1], [26], [38], but have not manipulated it consistently. To our knowledge, only one study [3] manipulated the respiration characteristics by increasing the *resistance* to inspiration during wheelchair propulsion and observed no change in respiratory and propulsion frequencies, or in the ratio between the two. In the present study, the pacing of propulsive or respiratory frequencies by a metronome set at the preferred (cycling or breathing) frequency was found to alter the coupling between breathing and cycling. This result suggests a bi-directionality in the coupling between the two systems, but the locomotor frequency appeared to drive the respiratory rhythm in greater proportion than inversely.

Interestingly, we noticed a change in the frequency ratios landscape particularly when participants were instructed to cycle in synchrony with the tone, in the form of a transition from higher-level Farey ratios to a lower Farey ratio (i.e. 1/2), a gain of stability for the 1/2 Farey ratio, accompanied by an increase in all criterion used to assess LRC stability. This crucial observation reinforces the importance to assess the complete ratio distribution and not only the mean ratio of the two frequencies [1]. Moreover, we observed an asymmetry in the frequency ratios landscape (see Figure 6) resulting probably from the low-to-moderate exercise intensity imposed to participants. The energetic constraint of the task did not require higher respiratory frequencies in order to furnish the needed oxygen to the muscles. The increase of the load during the task resulted in an increase in respiratory frequency without large modification of the pedaling frequency. As a consequence, a shift from the left half to the right half of the Farey tree near the 1/1 ratio should be observed. Finally, we observed a significant decrease of the respiratory frequency variability with the sound concomitant to the increase in %FRmode, irrespective of the synchronization conditions. This observation evidences a stabilization of the respiratory frequency when participants cycled in synchrony with a rhythmic external auditory stimulation [25] and confirms that breathing more regularly increases the degree of coordination between respiration and locomotor rhythms [15].

The results analyzed above – stabilizing effect of the sound, asymmetry, change with sound in the Farey tree dynamics, increase in stability – are very much in line with the music-at-sport and music-at-work literatures [39], [40], [41] and potentially predict why pacing movement frequency with music of similar rhythm can improve performance at work or in sport in long duration exercises. However, the assessment of how the respiratory system affects the coupling between breathing and moving remains to be documented in the music field. Our findings open new issues concerning the use of auditory stimulation during long duration physical activity and need to be confirmed in different sports using different music types. For physical activities implying stronger mechanical constraints than cycling, such as for instance in rowing, an attenuated effect of auditory stimulation on LRC due to more frequent natural LRC occurrences is expected. Conversely, for physical movements involving less mechanical constraints than cycling, music is susceptible to increase LRC occurrence and consequently decrease VO_2_ (see section 3.3). Interestingly, we observed two different adaptations to the auditory stimulation (see Figure 5 for an example), depending on the intrinsic LRC stability. High initial LRC stability was (negatively) affected by the sound (3 participants), and low-to-moderate initial LRC stability was increased by the sound (12 participants). Why were some participants initially more stable than others? One potential explanation is the proprioceptive anchoring effect [42]. The anchoring effect can occur without external stimulus [23] in the case of self-rhythmic oscillatory pronation or supination wrist movements, with a more pronounced stabilization for pronation [6]. This suggests that mechanical or neuromuscular constraints are involved in the anchoring phenomenon. In our pedaling task, a forcing point in the cycle exists where one has to exert the maximum force. This mechanical anchoring point could have been detected by some participants and used to improve their pedaling regularity. Certain participants were probably more tuned to this mechanical anchoring point than others. Some participants used their bike to come to work everyday, and it plausible that they have developed this specific perceptual attunement to the anchoring point. In any case, our results suggest the existence of a threshold of intrinsic LRC below which rhythmic sound is detrimental to the coupling between breathing and cycling, and above which sound positively influences LRC. More generally, the positive effect of external auditory information on LRC depends on its initial stability, as demonstrated by the linear and negative relation found between initial LRC stability and its sound-induced stabilization. This last point leads us to think that the auditory information effect is probably limited to the expert population known to exhibit more stable LRC than novices [15].

To the mechanical and neurological origins of LRC, we thus have to add an informational origin, exploiting (in our case) auditory and kinesthetic perceptual channels, acting as a LRC modulator. However, the contribution of these different components to the observed LRC is difficult to assess. One interesting perspective would be to manipulate these properties and evaluate their respective influence on coupling strength in a variety of physical activities.

### Energy Efficiency

Concerning the ventilatory parameters, our expectation derived from the Farey tree analysis was a significant reduction in VO_2_ concomitant to the sound-induced stabilization of LRC. Given that participants had to maintain the power output PAT at all time, a reduction of VO_2_ would reflect higher energy efficiency. On average, the value of VO_2_ in the Sound condition corresponded to a decrease around 4% when compared to its value with no sound. Our results demonstrated a significant reduction in oxygen consumption from 2.36 l.min to 2.32 l.min when participants were instructed to breathe or cycle in synchrony with the metronome, accompanying the improvement in LRC stability. This result confirms previous findings [2], [17] having shown a decrease in VO_2_ during periods of strong coupling, but with a more powerful methodology defining precisely the periods of synchronization. However, our results are in contradiction with other observations [18], [19], [20], [21] that did not find any change in VO_2_ due to coordination. In our case, the decrease in VO_2_ with sound should be the result of a better mobilization during cycling of the muscles involved in respiration, which are known to mechanically affect LRC [1], [43]. Our observation of an increase in both LRC and respiratory frequency when participants cycled in synchrony with the metronome supports that explanation. In our cycling task, locomotion facilitated the work of the respiratory system by mobilizing common muscles and thus reduced the energetic cost of respiration [44]. This decrease in VO_2_ with rhythmic sound is particularly interesting for amateur athletes practicing a sport at intensity below the anaerobic threshold (e.g., footing, cyclotouring, marathon, *etc.*). One interesting perspective is to manipulate the workload imposed to the participants in order to assess the efficiency of the auditory stimulation at more constrained intensity. Another avenue for research concerns the characteristics of the auditory stimulation presented to the participants. Our results demonstrate the efficiency of a periodic metronome set at the individual preferred frequency during steady-state exercises. However, in most cases, athletes need to modulate their behavior and be adaptive to continuously changing energetic demands (during cycling uphill or downhill for instance). In our future research, we plan to test the effect of endogenous auditory information, directly triggered from the movement of the participants. In line with perception-action and embodied perception theories, we expect auditory information to reinforce the participant’s perception of his/her own action, contributing to a better regulation of the ongoing movement with direct consequences for the stabilization of LRC.

In the field of bimanual coordination, a double-metronome situation in which each finger reversal (flexion and extension) occurred simultaneously with the auditory stimulus resulted in a significant increase of the local and global stabilization of coordination pattern when compared to a single-metronome condition [22]. It is conceivable that this type of double stimulation also increases more LRC stability and contributes to a larger diminution in oxygen consumption. This assumption derives from existing studies conducted on music and motor performance, showing that humans hearing music (which has a richer spectrum than a metronome) are able to maintain their effort for a longer period of time [39], [40]. However, it remains unclear whether these effects are solely due to the rhythm of the music, or whether other factors such as music frequency, melody, or personal experience play a role as well (for a review, see [45]).

### Conclusion

The Locomotor-Respiratory Coupling is a universal phenomenon underlying the production and supply of energy. Rhythmic activities such as walking, running, swimming, or rowing all exhibit LRC. However classical work points out the difficulty to describe LRC through the simple observation of frequency mode locking. Our results confirm this difficulty. Discrepancies in the results published so far on LRC are due to a lack of consensus about the definition and the methodology to investigate it. Here we extended the methodology adopted by previous authors [14] to the use of complex frequency mode locking patterns, associated with their relative phase dispersion, to express the strength of coupling between locomotion and respiration. Our results showed an increase in LRC and a corresponding decrease in energy consumption when participants were instructed to cycle in cadence with an auditory stimulation whose rhythm corresponded to the preferred locomotor frequency. The use of a metronome can be a useful way to postpone fatigue in exercises of long duration. Our current work imposes more constrains to participants in tasks such as running or cycling, reinforcing the stabilizing effect of the sound and increasing efficiency. Finally, another direction currently under investigation is the exploration of richer musical rhythms on LRC and energy expenditure.
